# Mycorrhizal Colonization Modulates the Essential Oil Profile and Enzymatic and Non-Enzymatic Antioxidants to Mitigate the Adverse Effects of Water Deficit in *Salvia* subg. *Perovskia*

**DOI:** 10.3390/biology11121757

**Published:** 2022-12-02

**Authors:** Mahvash Afshari, Mehdi Rahimmalek, Mohammed R. Sabzalian, Antoni Szumny, Adam Matkowski, Anna Jezierska-Domaradzka

**Affiliations:** 1 Department of Agronomy and Plant Breeding, College of Agriculture, Isfahan University of Technology, Isfahan 84156-83111, Iran; 2 Department of Horticulture, College of Agriculture, Isfahan University of Technology, Isfahan 84156-83111, Iran; 3 Department of Food Chemistry and Biocatalysis, Wrocław University of Environmental and Life Sciences, 50-375 Wrocław, Poland; 4 Department of Pharmaceutical Biology and Biotechnology, and Botanical Garden of Medicinal Plants, Wroclaw Medical University, 50-556 Wrocław, Poland

**Keywords:** gas chromatography, plant stress response, *Perovskia*, arbuscular mycorrhizal fungi, drought stress, water deficit, essential oils

## Abstract

**Simple Summary:**

We explored the modulating effect of arbuscular mycorrhizal fungi symbiosis on the water deficit stress symptoms in two medicinal and aromatic plants—*Salvia abrotanoides* and *S. yangii*. The essential oil content and composition, along with the enzymatic and non-enzymatic antioxidants, were studied to discover that the mycorrhizal fungi inoculation alleviated the deleterious impacts of water stress and influenced significantly the composition and content of essential oils. These effects may be explained by improved phosphorus uptake, chlorophyll biosynthesis, relative water content, and enzymatic and non-enzymatic antioxidant activities. Taken together, these findings highlighted the role of symbiosis with mycorrhizal fungi in increasing the tolerance to water deficit stress in medicinal and aromatic plants.

**Abstract:**

Among traditional Iranian herbs, *Perovskia* species (a subgenus of *Salvia*), while being valued ornamentals, are also studied for numerous potential pharmacological and therapeutic aspects. The current study was conducted to assess the effectiveness of two species of arbuscular mycorrhizal fungi (AMF), *Funneliformis mosseae* and *Rhizophagus intraradices,* separately and in combination, in terms of the essential oil content and compositions along with the enzymatic and non-enzymatic antioxidants in *Salvia abrotanoides* and *S. yangii* in response to three levels of irrigation, including 100% FC as well-watered, 75% FC (moderate irrigation deficit), and 50% FC (severe irrigation deficit). In both species, essential oil content, enzyme antioxidant activities, total phenolics, and flavonoids were increased significantly with the severity of stress; this increase was more pronounced in mycorrhizal inoculated herbs. Furthermore, leaf phosphorus concentration, relative water content, chlorophylls *a* and *b*, and total carotenoids decreased in parallel with reducing soil moisture; albeit, AMF inoculation improved the stress symptoms under increasing severity of water restriction compared with their control conditions. In addition, the percentage of root colonization was positively correlated with the relative water content (RWC) and leaf phosphorus concentration. Taking into account the essential oil groups, AMF colonization elevated some essential oil components, such as oxygenated monoterpenes, 1,8-cineol, camphor, and borneol, whereas the main sesquiterpenes, including E-β-caryophyllene and α-humulene, remarkably decreased. Taken together, these findings highlighted the role of symbiosis with AMFs in increasing the tolerance of water deficit stress in *S. abrotanoides* and *S. yangii* and improving their essential oil composition.

## 1. Introduction

Medicinal plants of the family Lamiaceae contain various terpenoid derivatives which render protective biological effects [1]. Among ornamental Iranian herbs, *Salvia* subg. *Perovskia* belongs to a unique example due to numerous potential therapeutic aspects. Until recently, some species of *Salvia* subg. *Perovskia* were considered to represent a separate genus, and its sister taxon is *Rosmarinus*. However, this genus was recently reclassified as the *Perovskia* subgenus of the extended genus of *Salvia* [2]. The current valid species names in the Kew database are *Salvia yangii* B.T. Drew (previously *Perovskia atriplicifolia*) and *Salvia abrotanoides* (Kar.) Sytsma (previously *P. abrotanoides*) [3]. Three species of the *Perovskia* subgenus have been identified in *Flora Iranica*, namely *P. abrotanoides* Karel., *P. artemisoides* Boiss., and *P. atriplicifolia* Benth., but *P. artemisoides* has not been recognized as a valid taxon [3,4]. *S. yangii* is also a popular ornamental species worldwide; its intensely scented leaves and flowers are used as a spice.

The various phytochemical constituents of the subgenus *Perovskia* are diterpenoids, rosmarinic acid, and other hydroxycinnamic acid derivatives, as well as monoterpenoids in essential oils (EOs). The numerous pharmacological effects of the *Perovskia* subgenus have been reported, such as anti-diabetic, antioxidant, antiplasmodial, cytotoxic to cancer cells, and antimicrobial and anti-inflammatory activities [5].

EO is a concentrated heterogeneous mixture of volatile constituents, including terpenoids (monoterpenes and sesquiterpenes), phenylpropanoids, and other aromatic compounds (chemically belonging to aldehydes, alcohols, ketones, esters, ethers, oxides, and some other compounds, usually specific to taxonomic groups). Monoterpenes and sesquiterpenes render characteristic aroma and biological properties to the EOs; hence, they are extensively used as flavoring and fragrance agents in food industries, pharmaceuticals, and cosmetics [6]. Terpenoids are the major class of specialized metabolites in *S. abrotanoides* [7,8]. The EOs are characterized by the presence of a large proportion of monoterpenes (70–80%) and a lesser proportion of sesquiterpenes (20–30%) [5]. The main biologically active constituents of *Perovskia* EO include camphor, 1,8-cineole, and bornyl acetate [5]. The contents of camphor and 1,8-cineole as main oxygenated monoterpenes of *Perovskia* EOs were, respectively, reported at 28.38% and 23.18% by Arabi et al. [9], 24% and 28% by Rustaiyan et al. [10], and 9.1% and 32.4% by Sajjadi et al. [11].

The synthesis of different secondary metabolites varies significantly depending on environmental and ecological factors and the type of plant material. Plants exposed to particular stress conditions generally produce higher concentrations of specialized secondary metabolites than non-stressed ones. More generally, water deficit stress is one of the multidimensional abiotic stresses that imposes profound effects on the quality and quantity of EOs along with the accumulation of bioactive secondary metabolites in aromatic plants, which in turn affects the biological efficacy of plants [12]. Reports in the literature indicated that successful and efficient use of deliberate irrigation regimes could directly increase the production of pharmaceutically desirable plant compounds [13].

In a sustainable agricultural system, the application of useful microorganisms, especially the arbuscular mycorrhizal fungi (AMF) group of soil microorganisms, is particularly important in maintaining soil quality and enhancing crop productivity [14]. AMF facilitates plant development by improving the capture of nutrients (particularly phosphorus) and improving resistance to abiotic stresses, including drought [15], high temperature [16], and salinity [17]. The beneficial influence of mycorrhiza is also manifested by an osmotic adjustment that can modify phytochemical profiles, for example, volatile compounds or polyphenols, as well as the enzymatic and non-enzymatic antioxidant defense network [18]. The effectiveness of AMF in upregulating the production of specialized metabolites during soil water storage reduction has been reported in several medicinal plants, such as *Cuminum cyminum* [19], *Pelargonium graveolens* [20], *Ocimum gratissimum* [21], and *Lallemantia iberica* [22]. The mechanism for increasing tolerance of water deficit by AMF is not fully understood. However, inoculation with AMF can enhance water absorption as a result of extraradical hyphae surrounding the plant roots followed by stomatal regulation. Changing the quality and quantity of EOs in medicinal herbs as a consequence of AMF treatment has been related to an alteration in the phytohormonal homeostasis, such as the level of cytokinins, gibberellins, and auxins, as well as to changes in the secretory structures, such as glandular trichomes. Additionally, the alleviation of stress conditions by AMF depends both on the host plant and on the species of mycorrhiza [12].

To the best of our knowledge, there are no previous reports on the simultaneous effects of water deficit and AMF on the *Salvia* subgenus *Perovskia*. Moreover, the alleviation of drought stress damage by AMF inoculation has not been studied in these medicinal plants. Therefore, we have aimed to (1) assess the effectiveness of AMF inoculation in alleviating the adverse effects of water deficit stress on two *Perovskia* species, (2) compare the bioameliorative impacts of two AMF fungal species (*Funneliformis mosseae* and *Rhizophagus intraradices*) separately and in combination under well-watered and stress conditions; and (3) explore the interactive effect of water deficit and AMF symbiosis on essential oil content and composition along with the enzymatic and non-enzymatic antioxidants in *S. abrotanoides* and *S. yangii*.

## 2. Materials and Methods

### 2.1. Plant Material

Plant materials consisting of two *Perovskia* species, *S. abrotanoides* and *S. yangii,* were collected from two provinces of Iran (Table 1). The plant specimens were identified based on *Flora Iranica* [23] by Dr. M. Rahimmalek (Isfahan University of Technology) and deposited in the herbarium of Isfahan University of Technology, Isfahan, Iran.

Climatic data were obtained from the Forests, Range, and Watershed Management Organization of Iran available on the Internet at http://www.rifr-ac.ir (accessed on 1 October 2022).

### 2.2. Experimental Design and Treatments

**Figure 1:**
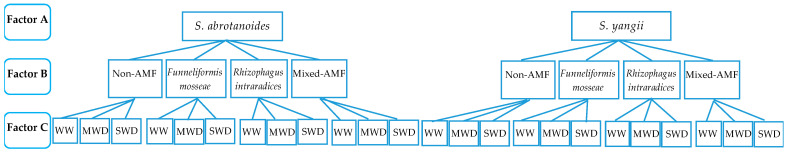
The schematic representation of the experimental design used in this study. WW = well-watered, MWD = moderate water deficit, SWD = severe water deficit. Non-AMF = without inoculation with arbuscular mycorrhizal fungi.

The mature seeds were sown in plastic pots of a 25.5 cm diameter and a 25 cm depth, each containing about 10 kg autoclaved (0.11 Mpa, 121 °C, 2 h) soil. The soil of a trial plot was composed of 67.9% silt, 7.2% clay, and 24.8% sand; phosphorus (P) 8.5 and potassium (K) 123 mg/kg; EC 1.5 dS/m; and pH 7.3. Initially, the pots were placed under controlled greenhouse conditions with 15/9 h light/dark cycles and ambient temperatures. The experiment was arranged based on a 2 × 3 × 4 factorial experiment established as a randomized complete block design, with three factors consisting of plant species, irrigation levels, and AMF inoculation with four replications and four individual plants per pot. Two AMF fungal species, namely *Glomus intraradices* (the new name is *Rhizophagus irregularis*), *Funneliformis mosseae*, and a mixture of both species supplied by the Turan Biotech Co., Shahroud, Iran, were utilized. Non-inoculated plants served as control samples. The mycorrhizal inocula comprised a mixture of spores (approximately 25 spores per gram) and were counted manually using a light microscope before the experiment. Specifically, 100 g of the prepared inoculum was placed below the seed in each pot and covered with soil. All treatment pots were well-watered for 3 months to establish roots, after which the various irrigation regimes were initiated. Three levels of irrigation, including 100% of the field capacity (FC) (no water deficit, further referred to as well-watered), 75% FC (moderate deficit irrigation), and 50% FC (severe deficit irrigation), were applied during the experiment. The irrigation volume was measured based on estimating the evapotranspiration (ET_C_) of the herb as follows: ET_C_ = ET_0_ × K_C_, where K_C_ is considered as the crop coefficient for the plant and ET_0_ is the reference evapotranspiration (mm d^−1^) calculated by the Penman–Monteith method [24]. The experiment was conducted for 25 weeks. Thereafter, the fresh aerial samples were harvested at the full-flowering stage and air-dried in shade at room temperature (25 °C ± 1). The scheme of the experimental design is illustrated in Figure 1.

### 2.3. Measured Parameters

#### 2.3.1. Percentage of Root Colonization by AMF

The fresh root systems were washed with KOH (10% *w/v*) at 95 °C for 1 h, acidified with HCl (1% *w/v*) for 1 h, and then stained with 0.05% *w/v* trypan blue in lactoglycerol (8:1:1 lactic acid, glycerol, and water) for 20 min. Finally, the percentage of mycorrhizal root infection was measured according to the gridline intersection method of Alipour et al. [25].

#### 2.3.2. Determination of Total Phenolics and Flavonoids

The stock solutions were prepared based on the method described previously by Rahimmalek et al. [1]. The total phenolic content in the methanolic extracts was determined using the Folin–Ciocalteu reagent with tannic acid as the external standard. The final results are reported in mg of tannic acid equivalent per g dry weight of the sample (mg TAE g^−1^ DW) [26]. Estimating total flavonoid content was performed using the aluminum chloride colorimetric technique following the method of Toor and Savage [27], with quercetin as a reference standard. Total flavonoid content was expressed as mg quercetin equivalent per g dry weight of the sample (mg QUE g^−1^ DW).

#### 2.3.3. Antioxidant Enzyme Activities

The ascorbate peroxidase (APX) and guaiacol peroxidase (POD) enzyme activities in leaf extracts were examined, as described by Nouraei et al. [13]. An amount of 0.2 g of fresh plant tissue was homogenized in the sterile vial with a 1 mL extraction buffer (containing 2 mM α-dithiothreitol, 2 mM EDTA, 0.2% Triton X-100, 50 mM Tris-HCl, and 2% polyvinylpyrrolidone) and was centrifuged for 30 min at 4 °C. The supernatant was used to measure the activity of all enzymes. All steps were carried out at 4 °C. For the determination of ascorbate peroxidase activity, a 3 mL extraction buffer, 4.51 μL H_2_O_2_ (30%), 100 μL of 5 mM ascorbate, and 50 μL of extraction solution were mixed. The absorbance was read at 290 nm every 30 s for 2 min. For the assessment of guaiacol peroxidase activity, an extraction buffer of 3 mL, 4.51 μL H_2_O_2_ (30%), 3.35 μL Guaiacol, and 50 μL of extraction solution were mixed together. The absorbance was determined at 470 nm every 30 s for 2 min. Enzyme activities were calculated as micromoles of decomposed per milligram of protein per minute (µmol min^−1^ mg^−1^ protein). The total protein level of the extracts was measured at 595 nm using the Bradford method [28].

#### 2.3.4. Leaf Phosphorus Concentration and Relative Water Content

To quantify the concentration of leaf phosphorus (P), approximately 1 g of dried leaf samples were separately ground and incinerated for 5 h at 550 °C in a muffle furnace. The digestion procedure was performed in a water bath at 28 °C for 25 min by adding 10 mL of hydrochloric acid (2N) into a 100 mL volumetric flask. The mixtures were filtered using filtration paper (Whatman No. 42, Whatman Corp. Little Chalfont, UK) and then diluted to 100 mL with the addition of distilled water. The amount of leaf phosphorus was measured spectrophotometrically at 880 nm, according to the vanadate-molybdate phosphoric acid method [29]. The standard curve was prepared by taking 0 to 1 mg/L of P concentrations. The samples were diluted based on the standard curve. The relative water content (RWC) was assayed in leaf samples according to the method outlined by He et al. [30]. Sampling was carried out an hour after solar noon. Six top fully expanded leaves from different plants per replication were sampled with a sharp knife and immediately used. The leaves were weighted (FW) and then were hydrated in distilled water for 24 h at room temperature. Turgid samples were quickly weighted (TW) after drying surface water with paper towels. Dry weights (DW) were measured after drying at 75 °C for 48 h. RWC was calculated according to the following equation:

RWC (%) = [(FW − DW)/(TW − DW)] × 100

#### 2.3.5. Chlorophyll and Carotenoid Contents

The contents of chlorophylls *a* and *b* and total carotenoids were quantified by the method proposed by Arnon [31]. The absorbance of the solutions was recorded at wavelengths 470 nm for carotenoids along with 645 and 663 nm for chlorophylls. Chlorophylls and total carotenoids were expressed as mg g^−1^ dry weight. For photosynthetic pigment determination, 0.3 g of a fresh leaf was homogenized and extracted in 10 mL acetone 80% using the centrifuge run at 3000 rpm for 15 min. Concentrations (mg/g FW) of chlorophylls *a* and *b,* as well as carotenoids, were calculated according to the following formulas:

$$\mathrm{Chlorophyll}\;\left(a\right)=\frac{\left(19.3\times A663\right)-\left(0.86\times A645\right)\times V}{100W}$$

$$\mathrm{Chlorophyll}\;\left(b\right)=\frac{\left(19.3\times A645\right)-\left(3.6\times A663\right)\times V}{100W}$$

$$\mathrm{Carotenoids}=\frac{\left[\left(1000\times A470\right)-\left(3.27\times \mathrm{mg}\;\mathrm{Chl}\;\left(a\right)\right)-\left(104\times \mathrm{mg}\;\mathrm{Chl}\;\left(b\right)\right)\right]}{227}$$

where A663, A645, and A470 represent absorbance values of the pigments at λ = 663, 645, and 470, and V and W are extract volumes after filtration and fresh weight of the leaf sample, respectively.

#### 2.3.6. Essential Oil Content

A total of 60 g of the powdered samples was subjected to hydro-distillation in 400 mL of water and run for 5 h using the round-bottom flask (a volume of 1000 mL) of a Clevenger-type apparatus. EO content (%) was calculated as a relative percentage basedon the following equation [6]:

$$\mathrm{EO}\;\mathrm{content}\;(\%)=\frac{\mathrm{mass}\;\mathrm{of}\;\mathrm{obtained}\;\mathrm{EO}\;\left(g\right)}{\;\mathrm{mass}\;\mathrm{of}\;\mathrm{dry}\;\mathrm{matter}\;\left(g\right)}\times 100$$

#### 2.3.7. GC–FID and GC/MS Analyses

EO constituents were determined by GC using an Agilent model 7890 gas chromatograph equipped with a DB-5 fused silica column (film thickness 0.25 μm, 30 m length × 0.25 mm diameter) as well as an Agilent model 5975 as GC–MS/FID detection was monitored at 70 eV with a mass range of 39 to 400 *m/z*. The oven temperature was programmed as follows. The initial temperature of 60 °C was maintained for 4 min and immediately increased to 220 °C at the rate of 3 °C/min; subsequently, the temperature was elevated to 260 °C at the rate of 20 °C/min and held at this temperature for 10 min. Detector (FID) and injector temperatures were programmed at 280 °C. Nitrogen was used as a carrier gas source with a linear velocity of 0.7 mL/min.

#### 2.3.8. Essential Oil Components Identification

The volatile compounds were quantified by comparing their experimentally calculated retention indices (RI) relative to C_8_–C_24_ *n*-alkanes and were injected into the DB-5 column in the same condition. Furthermore, their spectral mass was matched with the internal reference mass spectra databases, including Willey 275 (Chem Station data system) and NIST 08 (the National Institute of Standards and Technology). Quantitation of EO constituents was conducted by the respective FID area under the curve using the peak-area normalization and neglecting response factors [5].

### 2.4. Data Analysis

All measured parameters were subjected to a three-way analysis of variance (ANOVA) using the SAS statistical software (version 9.4, SAS Institute, Cary, NC, USA). The analysis of variance (ANOVA) related to the studied traits under different irrigation regimes and AMF inoculation in *Perovskia* species was indicated in Table 2. Furthermore, significance of differences in the irrigation regime × AMF (each strain separately and mixed) × plant species interactions have been indicated in Table 3. The principal component analysis (PCA) and correlation coefficients were carried out using GraphPad software (Prism version 9.0, San Diego, CA, USA).

## 3. Results

### 3.1. Analysis of Variance

The results of ANOVA revealed that the main effects of the irrigation regime, AMF, plant species, and their interactions were statistically significant (*p* ≤ 0.01) on total phenolic and flavonoid contents, ascorbate peroxidase, guaiacol peroxidase, phosphorous concentration, relative water content, chlorophylls *a* and *b*, and carotenoid, as well as essential oil content (Table 2). Moreover, the first-order interactions (irrigation regime × AMF, irrigation regime × plant species, and AMF × plant species) significantly influenced all studied traits except for the effect of irrigation regime × plant species on POD. The results showed that the second-order interaction (irrigation regime × AMF × plant species) had a significant effect on all traits (Table 2).

### 3.2. Root Colonization Percentage

**Figure 2:**
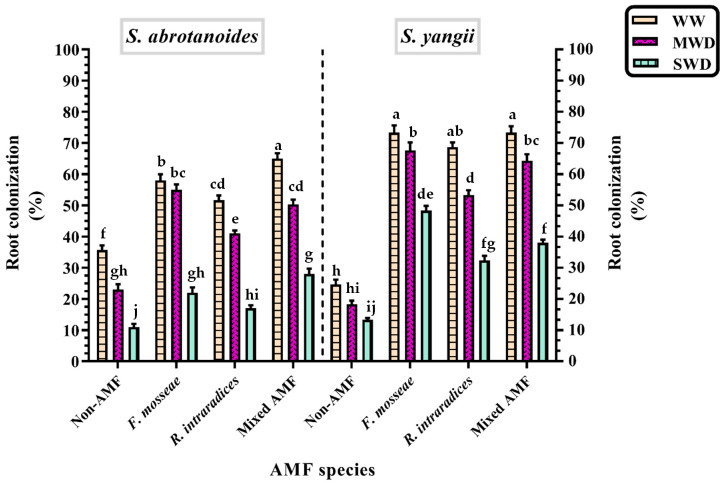
The root colonization (%) by AMF in *S. abrotanoides* and *S. yangii* under different irrigation regimes. Means ± SD with seven repetitions. Different small letters above the bars indicate a significant difference at *p* ≤ 0.05.

**Figure 3:**
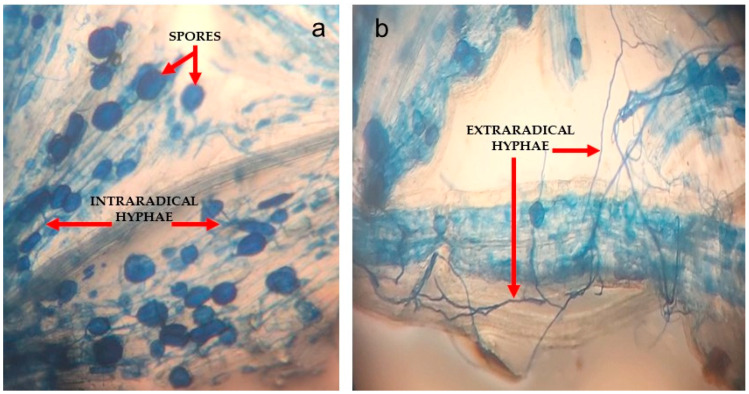
Symptoms of the establishment of a symbiotic relationship among AMF and the root of *Salvia abrotanoides* (×10 objective lens); (**a**) spores and intraradical hyphae; (**b**) extraradical hyphae. The fresh root systems were stained with 0.05% *w/v* trypan blue in lactoglycerol (8:1:1 lactic acid, glycerol, and water).

The present study revealed that the application of AMF significantly elevated the colonization percentage of the inoculated samples irrespective of irrigation regimes (Figure 2 and Figure 3) compared with their corresponding non-AMF samples in both plant species. As shown in Figure 2, the percentage of root colonization was decreased by reduced soil moisture in *S. yangii* and *S. abrotanoides*. Compared to the controls, water deficit stress decreased root mycorrhizal colonization by 2.76 times in *S. abrotanoides,* as well as 1.50 times in *S. yangii* (Figure 2).

Nevertheless, the highest root colonization was recorded in plants irrigated optimally and inoculated with mixed AMF in *S. abrotanoides* and *S. yangii* with values of 66% and 74%, respectively. In *S. yangii* inoculated with *F. mosseae*, the root colonization is exactly the same as that of mixed AMF-inoculated *S. yangii* (Figure 2). The lowest values were measured in non-AMF samples, followed by severe stress conditions in *S. abrotanoides* (11%) and *S. yangii* (13%). Inoculation with *F. mosseae*, *R. intraradices,* and mixed AMF increased the percentage of root colonization by 2.33, 1.71, and 2.04 times in *S. abrotanoides,* and 3.58, 2.79, and 3.42 times in *S. yangii* in comparison to plants without AMF, respectively. Thus, *S. yangii* displayed a higher root colonization percentage than *S. abrotanoides* across all treatments (Figure 2). Moreover, *F. mosseae* was more effective in root colonization in both plant species than the other ones, regardless of irrigation regime (Figure 2).

### 3.3. Total Phenolics and Flavonoids

Total phenolics and flavonoids increased with reduced soil moisture in both *S. yangii* and *S. abrotanoides*, regardless of the irrigation treatments. Compared to the controls, water deficit stress increased total phenolics and flavonoids by 1.10 and 1.57 times in *S. abrotanoides,* and 1.51 and 1.23 times in *S. yangii*, respectively (Table 3). The application of AMF dramatically elevated leaf phenolics and flavonoids in all irrigation regimes (Table 3) compared to their corresponding non-AMF samples in both plant species. The highest amount of total phenolics was recorded in plants inoculated with *F. mosseae,* followed by severe stress conditions in *S. abrotanoides* and *S. yangii,* with values of 201.95 and 393.97 mg TAE g^−1^ DW, respectively. The lowest values were measured in optimally irrigated plants and non-AMF samples in *S. abrotanoides* (94.65 mg of TAE g^−1^ DW) and *S. yangii* (111.22 mg of TAE g^−1^ DW). Inoculation with *F. mosseae*, *R. intraradices,* and mixed AMF increased the leaf phenolic content by 1.46, 1.21, and 1.30 times in *S. abrotanoides,* as by well as 2.11, 1.13, and 1.05 times in *S. yangii* compared to the non-AMF plants, respectively. A similar trend was displayed for the accumulation of flavonoids in both *Perovskia* species. The highest flavonoid content was observed in samples inoculated with *F. mosseae* in both plant species of *S. abrotanoides* (12.59 mg QUE g^−1^ DW) and *S. yangii* (14.32 mg QUE g^−1^ DW), responding to severe stress conditions (Table 3). Inoculation with *F. mosseae*, *R. intraradices*, and mixed AMF increased the leaf flavonoid content by 1.49, 1.33, and 1.40 times in *S. abrotanoides,* as well as 1.53, 1.34, and 1.13 times in *S. yangii* in comparison to plants without AMF, respectively (Table 3).

### 3.4. Enzymatic Antioxidant Defense Systems

According to the mean comparison of treatments, the enzymatic antioxidant activities were increased by reducing soil moisture in both *S. yangii* and *S. abrotanoides*. Compared to the controls, water deficit stress elevated APX and POD activity by 2.40 and 2.63 times in *S. abrotanoides,* and 1.70 and 2.04 times in *S. yangii*, respectively (Table 3). Consequently, *S. yangii*, as a drought-tolerant species, exhibited more antioxidant-defensive response ability with increasing water deficit intensity than the other species.

The application of AMF considerably elevated leaf peroxidase activities under all irrigation regimes (Table 3) compared with their corresponding non-AMF samples in both plant species. The highest amount of APX activity was recorded in plants inoculated with *F. mosseae*, followed by severe stress conditions in *S. abrotanoides* and *S. yangii,* with values of 862.67 and 1044.66 μmol min^−1^ mg^−1^ protein, respectively (Table 3). The lowest values were measured in plants irrigated optimally and non-AMF samples in *S. abrotanoides* (181.79 mg μmol min^−1^ mg^−1^ protein) and *S. yangii* (279.28 μmol min^−1^ mg^−1^ protein). Inoculation with *F. mosseae*, *R. intraradices*, and mixed AMF increased the APX activity by 1.77, 1.52, and 1.25 times in *S. abrotanoides,* and 1.97, 1.48, and 1.19 times in *S. yangii* in comparison to plants without AMF, respectively. A similar trend was displayed for POD activity in both *Perovskia* species (Table 3). The highest POD activity was observed in samples inoculated with *F. mosseae* in both plant species of *S. abrotanoides* (72.38 mg μmol min^−1^ mg^−1^ protein) and *S. yangii* (86.38 mg μmol min^−1^ mg^−1^ protein), responding to severe stress conditions (Table 3). Inoculation with *F. mosseae*, *R. intraradices*, and mixed AMF increased the POD activity by 1.98, 2.31, and 1.89 times in *S. abrotanoides,* and 3.38, 2.21, and 2.02 times in *S. yangii* compared to the non-AMF plants, respectively (Table 3).

### 3.5. Leaf Phosphorus Concentration and Relative Water Content

According to the mean comparison of the treatments, P concentration and RWC were diminished in parallel with the reduction in soil moisture in both species. Compared to the controls, under water deficit stress, the P concentration and RWC decreased by 1.23 and 1.34 times in *S. abrotanoides,* and 1.22 and 1.25 times in *S. yangii*, respectively (Table 3).

Application of AMF significantly elevated leaf P concentration and RWC under all irrigation regimes compared with their corresponding non-AMF samples in both plant species in the following order: *F. mosseae* > *R. intraradices* > mixed AMF (Table 3). The highest P concentration was recorded in the plants irrigated optimally and inoculated with *F. mosseae* in *S. yangii* and *S. abrotanoides,* with values of 0.72 and 0.58 mg g^−1^, respectively. The lowest values were measured in non-AMF samples, followed by severe stress conditions in *S. abrotanoides* (0.34 mg g^−1^) and *S. yangii* (0.35 mg g^−1^). Compared to *S. abrotanoides*, leaves of *S. yangii* contained higher total phosphorus concentrations regardless of irrigation regimes or inoculation with AMF, indicating the efficiency of the roots of *S. yangii* to promote P uptake from soil. The highest RWC was observed in samples inoculated with mixed AMF in both plant species of *S. abrotanoides* (79.61%) and *S. yangii* (84.17%), responding to WW conditions (Table 3). Inoculation with *F. mosseae*, *R. intraradices*, and mixed AMF increased leaf RWC by 1.93, 1.83, and 1.64 times in *S. abrotanoides,* and 1.82, 1.71, and 1.62 times in *S. yangii* compared to the non-AMF plants, respectively (Table 3).

### 3.6. Photosynthetic Pigments

According to the mean comparison of treatments, chlorophyll *a*, *b*, and carotenoid contents are decreased by reducing the soil moisture in both *S. yangii* and *S. abrotanoides* species. Compared to the controls, water deficit stress decreased the chlorophylls *a*, *b*, and carotenoids by 1.87, 1.28, and 1.92 times in *S. abrotanoides,* and by 2.02, 1.50, and 1.84 times in *S. yangii*, respectively (Table 3). Although, the application of AMF significantly elevated leaf chlorophylls and carotenoids under all irrigation regimes (Table 3) compared to their corresponding non-AMF samples in both plant species in the following order: *F. mosseae* > mixed AMF > *R. intraradices*. The highest amount of chlorophyll *a* was recorded in plants irrigated optimally and inoculated with *F. mosseae* in *S. abrotanoides* and *S. yangii* with values of 20.48 and 17.01 mg g^−1^ FW, respectively. The lowest values were measured in non-AMF samples, followed by severe stress conditions in *S. abrotanoides* (4.50 mg g^−1^ FW) and *S. yangii* (2.85 mg g^−1^ FW). A similar trend was displayed for chlorophyll *b* content in both *Perovskia* species. The highest total carotenoids were also observed in samples inoculated with *F. mosseae* in *S. abrotanoides* (1.98 mg g^−1^ FW) and *S. yangii* (1.78 mg g^−1^ FW), responding to well-watered plants (Table 3). Inoculation with *F. mosseae*, *R. intraradices*, and mixed AMF increased the leaf carotenoids by 1.51, 1.28, and 1.36 times in *S. abrotanoides,* and by 1.76, 1.27, and 1.69 times in *S. yangii* in comparison to the non-AMF plants, respectively (Table 3).

### 3.7. Essential Oil Content

The EO content was remarkably dependent on water deficit treatments, AMF, and plant species (Table 4 and Table 5). The EO content was elevated with the severity of stress in both plant species (Table 4 and Table 5) in the following order: SWD > MWD > WW. However, this increase was more prominent for *S. abrotanoides*. In other words, water deficit stress-induced biosynthesis of EOs in both plant species, as compared with a well-watered irrigation regime.

Interestingly, all the inoculation treatments improved the EO content compared to the non-AMF treatment (Table 4 and Table 5) in both cases of water-stressed and control samples. The highest EO content was obtained in samples inoculated with *F. mosseae* in both plant species of *S. abrotanoides* (3.78%) and *S. yangii* (2.90%), responding to severe water stress levels (Table 4 and Table 5). The lowest EO content was recorded in plants irrigated optimally and un-inoculated *S. yangii* (0.61%) and *S. abrotanoides* (0.54%). Under the severe drought stress, inoculation with *F. mosseae*, *R. intraradices*, and mixed AMF increased the EO content by 2.22, 1.29, and 1.40 times in *S. abrotanoides,* as well as by 2.08, 1.56, and 1.44 times in *S. yangii* in comparison to the non-AMF plants, respectively.

### 3.8. Essential Oil Composition

The EO compositions depended on water deficit treatments, AMF, and plant species (Table 4 and Table 5). In total, 36 constituents were identified in *Perovskia* EOs, comprising 94.86–100% of total detected fractions (Table 4 and Table 5). In all treatments, the major compounds of both *Perovskia* species were 1,8-cineol (5.20 to 30.20%), followed by camphor (2.95 to 23.53%), bornyl acetate (2.10 to 12.93%), borneol (3.20 to 11.92%), E-β-caryophyllene (3.93 to 11.42%), α-humulene (3.26 to 10.39%), and δ-3-carene (0.15 to 10.21%). Based on the classification of detected terpenes, oxygenated monoterpene was a fundamental part of EOs compositions that ranged from 20.67 to 51.36% in *S. abrotanoides* and from 24.63 to 49.93% in *S. yangii* (Table 4 and Table 5).

Water deficit treatment induced biosynthesis of the monoterpenes (oxygenated monoterpenes and monoterpene hydrocarbons) in plant species in the following order: SWD > MWD > WW, as compared with a well-watered irrigation regime. Although, oxygenated sesquiterpenes decreased with the severity of stress in both plant species (Table 4 and Table 5) in the following order: WW > MWD > SWD. Considering EO groups, AMF colonized *S. abrotanoides* and *S. yangii* plants had elevated oxygenated monoterpenes: 1,8-cineol, camphor, and borneol, disregarding stress conditions; by contrast, the main sesquiterpenes, including E-β-caryophyllene and α-humulene, remarkably decreased (Table 4 and Table 5).

The highest amount of 1,8-cineol was obtained in samples inoculated with mixed AMF in both plant species of *S. abrotanoides* (20.34%) and *S. yangii* (30.20%), responding to severe water stress levels (Table 4 and Table 5). Under severe drought stress, inoculation with *F. mosseae*, *R. intraradices*, and mixed AMF increased the EO content by 1.51, 1.57, and 1.59 times in *S. abrotanoides,* and 1.52, 1.35, and 1.71 times in *S. yangii* in comparison to the non-AMF plants, respectively. The highest value of camphor was recorded in plants inoculated with mixed AMF followed by severe stress conditions in *S. yangii* (23.53%) and mild stress conditions in *S. abrotanoides* (15.21%). Furthermore, the content of δ-3-carene, as a monoterpene hydrocarbon was significantly increased by inoculation with AMF compared to the non-AMF samples. In *S. abrotanoides*, borneol ranged from 3.20% in SWD and non-AMF treatment to 11.92% in SWD and *R. intraradices* species, while it varied from 2.87% in SWD and non-AMF treatment to 8.48% in SWD and *F. mosseae* species in *S. yangii* (Table 4 and Table 5).

### 3.9. Correlation and Principal Component Analysis

**Figure 4:**
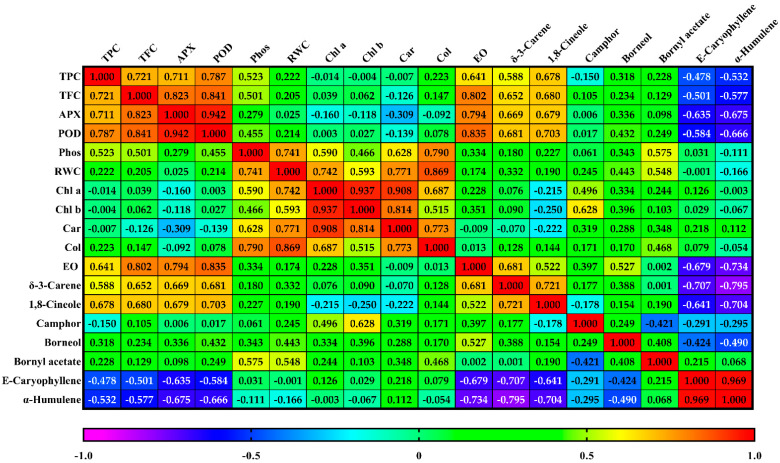
The heat map-based correlation analysis among all biochemical parameters under different irrigation regimes followed by AMF inoculation in *S. abrotanoides* and *S. yangii*.

Correlation analysis showed a significant and positive relationship among EO content with POD (r = 0.84 **), TFC (r = 0.80 **), APX (r = 0.79 **), δ-3-carene (r = 0.68 **), borneol (r = 0.53 **), and 1,8-cineole (r = 0.52 **). The strong positive correlation was firmly established among E-β-caryophyllene and α-humulene (r = 0.97 **), APX and POD (r = 0.94 **), chlorophylls *a* and *b* (r = 0.94 **), chlorophyll *a* and carotenoid (r = 0.91 **), as well as POD and TFC (r = 0.84 **) (Figure 4). 1,8-cineol was also found positively correlated with δ-3-carene (r = 0.72 **), POD (r = 0.70 **), TFC, TPC, and APX (r = 0.68 **), but was negatively correlated with α-humulene (−0.70 **) and E-β-caryophyllene (0.64 **). Clearly, the root colonization percentage was found to be positively correlated with RWC (r = 0.87 **), leaf phosphorus concentration (r = 0.79 **), carotenoid (r = 0.77 **), and chlorophyll *a* (r = 0.69 **). Moreover, the results indicated significant positive correlations between POD and APX with TFC and TPC (Figure 4).

**Figure 5:**
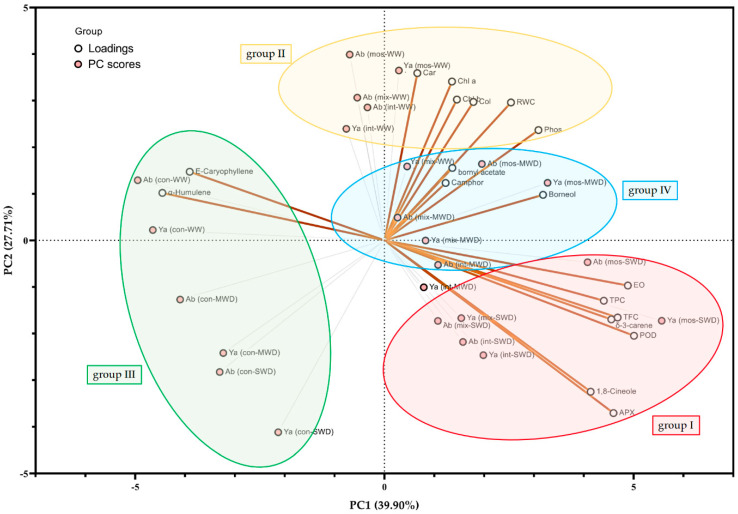
The principal component analysis (PCA biplot showing PC scores (mauve-filled circles) and loadings (white-filled circles) based on all biochemical parameters under different irrigation regimes followed by AMF inoculation in *S. abrotanoides* and *S. yangii*.

In general, PCA was performed on all studied chemical variables to show possible groupings between the samples and treatments, as indicated by the formation of four basic categories (Figure 5). According to the PCA dendrogram, most of the variation (67.61%) was described by the first two principal components, with the first followed by the second axes contributing to 39.90% and 27.71% of the sample’s variance, respectively (Figure 5). The first group comprised EO content, TPC, TFC, δ-3-carene, and enzymes (APX, and POD) (group I). The physiological characteristics containing chlorophylls *a* and *b*, carotenoid, bornyl acetate, RWC, leaf phosphorus concentration, and root colonization percentage fell into the same category (group II). In addition, the root colonization percentage was found positively correlated with RWC and leaf phosphorus concentration (Figure 5). Both plant species exhibited the highest main sesquiterpenes, including E-β-caryophyllene and α-humulene, in non-AMF samples irrespective of irrigation regimes (group III). In contrast, a negative relationship was observed among volatile monoterpenes and sesquiterpenes (Figure 5). Moreover, AMF-inoculated plants with all fungal species followed by mild stress conditions in *S. abrotanoides* and *S. yangii* were located in a discrete group (group IV) around the central part of the PCA biplot which was related to the middle average estimates of most parameters, such as percentage of camphor and borneol. AMF colonization elevated oxygenated monoterpenes, such as camphor and borneol, in both plant species under stress conditions (group IV).

## 4. Discussion

According to the results, a decrease in AMF colonization with the severity of stress could be due to the reduction in carbon availability in the host plants, soil microbial activity, colonization capacity of AMF, hyphal extension, as well as spore germination and development under water deficit conditions [30]. A negative correlation was obtained between AMF colonization and water deficiency in a wide range of plant species, including *TrifoChun-Yanm repens* [32], *Vigna mungo* [33], and *Ocimum gratissimum* [34]. Therefore, the optimal amount of available water is required for the survival and development of AMF in the soil [20]. Moreover, *F. mosseae* was more effective in root colonization in both plant species than the other ones, regardless of irrigation regime (Figure 2). Giovannetti et al. [35] found that the behavior of each AM fungi in soils could be different, even under the same conditions. In addition, the ability of fungal species to adapt to water shortages depends on both the severity and the duration of the stress.

Inoculation with *F. mosseae*, *R. intraradices*, and mixed AMF increased the leaf phenolic and flavonoid contents in *S. abrotanoides* and *S. yangii* in comparison to plants without AMF (Table 3). Amiri et al. [18] also reported a similar trend, indicating that *F. mosseae*, *R. intraradices*, and mixed AMF treatments were effective to ameliorate the adverse effect of water deficit on the phenolic and flavonoid contents in comparison to the non-AMF samples in *Pelargonium graveolens*, in which flavonoid content reached a peak when plants were inoculated by *F. mosseae*. As a part of the non-enzymatic antioxidant system, phenolic and flavonoid compounds play essential roles in protecting plants from excessive ROS production by quenching singlet oxygen, binding with ROS, breaking lipid peroxidation chain reactions, and chelating transition metals [36]. The current results were in line with other studies on other medicinal and aromatic plants such as *Pelargonium graveolens* [18], *Cumin cyminum* [19], *Melissa officinalis* [37], *Ocimum ciliatum,* and *Ocimum basilicum* [38], which reported rising polyphenolic contents corresponding to the severity of water deficit. The shikimic acid pathway is involved in the biosynthesis of a central significant portion of polyphenolic compounds using carbohydrate precursors. Therefore, improved carbohydrate metabolism in AMF-treated plants may stimulate the synthesis of phenolics and flavonoids or provide an energy source for AMF colonization [39]. AMF can produce various phytohormones and secondary metabolites, such as polyphenolic compounds in hosts, which contribute to the regulation of the growth, development, and adaptation of herbs. Therefore, the accumulation of phenolics and flavonoids in plants inoculated with AMF might be connected with diminishing oxidative damage mediated by ROS during water deficit conditions [18]. A similar trend of association between AMF inoculation and polyphenol accumulation causing the amelioration of stress severity level was observed in *Sesamum indicum* [14], *Ocimum gratissimum* [21], *Nicotiana tabacum* [40], and *Olea europaea* [41].

Inoculation with *F. mosseae*, *R. intraradices*, and mixed AMF increased the APX and POD activities in *S. abrotanoides* and *S. yangii* compared to the non-AMF plants (Table 3). Amiri et al. [18] also reported that *F. mosseae* was more effective in terms of APX and POD activities under water stress conditions in *Pelargonium graveolens* as compared with *R. intraradices*, mixed AMF, and non-AMF samples. The increase in both enzyme activities in response to levels of stress revealed an innate antioxidant defense mechanism in two different species of *Perovskia*. Similarly, the up-regulated activities of peroxidase and reduced accumulation of ROS under water deficit stress have been reported in *Cynara cardunculus* [13], *Thymus daenensis* [42], and *Coleus amboinicus* [43]. Higher enzymatic antioxidant activities in the AMF-treated plants than in the non-AMF samples indicate that AMF symbiosis can improve maintenance of a dynamic balance between ROS accumulation and elimination, thus reducing the oxidative damage, such as membrane lipid peroxidation, under water deficits [44]. The activation of the enzymatic protective system by mycorrhization is correlated with the increase in plant drought tolerance and free radical scavenging activities via enzymes. Furthermore, multiple unique genes that encode antioxidant enzymes can be induced by plants treated with AMF, whose expression patterns can stimulate antioxidant enzyme activities under drought stress conditions [45]. In fact, the extension of AMF hyphae and lateral roots can involve the transport of essential micronutrients throughout the development of a plant. The AMF-treated plants exposed to water deficit stress contained more active antioxidant enzymes than the non-AMF ones [46]. It is well documented that peroxidase enzyme activities increased in *Lallemantia iberica* [22], *Camellia sinensis* [44], *Glycyrrhiza glabra* [45], *Carum copticum* [46], and *Dracocephalum moldavica* [47] in the AMF-inoculated herbs exposed to water deficit stress. APX scavenges are potentially harmful H_2_O_2_ through ascorbate as an electron donor in the ascorbate-glutathione pathway. Overall, the enhancement of APX and POD activities in AMF-treated samples can be considered an intrinsic defense strategy in H_2_O_2_ degradation under drought-stress conditions [45].

Phosphorus concentration in the leaves can be influenced by the stomatal response to water deficit stress, wall stiffening regulating stomatal dynamics, or by impacting the energetics associated with guard cell osmotic properties [48]. The decline in leaf P uptake under stressful conditions in both species of *Perovskia* can be explained by reduced root expansion and phosphorous transfer into plants [14]. Mycorrhizal symbiosis can significantly contribute to increasing cell division and root proliferation under water deficit conditions. Moreover, improvement in the absorption of phosphorus in dry soils was probably due to the penetration of AMF mycelium into the rhizosphere, which allows the use of a larger soil volume via developing a complementary absorption system [22]. Davies et al. [49] suggested that AMF-treated plants can increase the P concentration by improving the leaf’s relative water content. These findings were in line with other previous research results on *Sesamum indicum* [14], *Lallemantia iberica* [22], and *Foeniculum vulgare* [48], which found that AMF inoculation improved P concentration under water deficit stress.

Leaf RWC is considered a reliable measure of biochemical indices and plant water status, as it can be helpful to estimate drought stress intensities. Water deficit stress induces stomatal closure, thereby reducing the transpiration rate, which leads to decreased plant growth and development. Meanwhile, stomatal conductance strongly depends on the water supply from the soil-root system as well as patterns of plant canopy evaporation [45]. Better leaf water status in the AMF-inoculated samples than in non-inoculated ones can be attributed to the high efficiency of root hydraulic conductivity along with water uptake from bulk soil into host herbs imparted by the external hyphae and improved stomatal and osmotic regulation based on hormonal adjustment [30]. The relative water contents in water deficit conditions were twice than in saturated soils in both plant species (Table 3). The achieved results are in accordance with earlier reports on *Thymus daenensis* [12], *Pelargonium graveolens* [18], *Poncirus trifoliata* [30], and *Camellia sinensis* [44]. They described the increment of RWC values in AMF-inoculated samples subjected to the well-watered and water deficit conditions.

The considerable reduction in the level of chlorophylls under water deficit stress could be interpreted by up-regulating chlorophyllase activity along with membrane lipid peroxidation or down-regulating other related enzymes responsible for chlorophyll biosynthesis, such as *Cab* gene families and, ultimately, chlorophylls decomposition in plant leaves [14]. Furthermore, chlorophyll *a* is more sensitive to water deficit than chlorophyll *b*, which is consistent with the results of the current study (Table 3). In this context, a similar trend of association between the extent of water stress and the reduction in photosynthetic pigments has been reported in *Cynara cardunculus* [13], *Ocimum ciliatum, Ocimum basilicum* [38], *Nigella sativa* [50], and *Anethum graveolens* [51]. The chlorophyll content can be considered as the suitable evaluation criterion in herbs; hence, its reduction act as the main limiting factor for photosynthesis under water deficit stress conditions [13]. Carotenoids are pigments with several functions contributing to the antioxidant defense of plants by quenching singlet molecular oxygen and dissipating excess excitation energy in protecting photosystems against water deficit stress conditions [39]. Decreased total carotenoids with reduced soil moisture stress may be due to the formation of xanthine in the xanthophyll cycle followed by the decomposition of beta-carotene [25]. AMF samples can accelerate the photosynthesis rate by the antagonistic effect of Na^+^ on chlorophyll biosynthesis, balance nutritional status, and improve phosphorus uptake efficiency, which is crucial for leaf CO_2_ assimilation [20]. Zhu et al. [52] suggested that AMF-treated plants can increase the expression of chlorophyll biosynthetic genes and reduce chlorophyllase activity, resulting in a greater synthesis of photosynthetic pigments. These findings were in line with other previous research results on *Pelargonium graveolens* [20], *Carum copticum* [46], *Ocimum basilicum* [53], *Lactuca sativa* [54], and *Antirrhinum majus* [55], which found that AMF inoculation improved chlorophylls and carotenoids biosynthesis with increasing severity of water restriction, as compared with their control conditions.

Water deficit stress-induced biosynthesis of EOs occurred in both of the studied plant species, as compared with a well-watered irrigation regime (Table 4 and Table 5). Similarly, several investigators revealed the highest level of EO contents facing severe water scarcity in some medicinal species, including *Thymus daenensis*, *Thymus vulgaris* [12], *Carum copticum* [46], *Nigella sativa,* and *Petroselinum crispum* [50]. Overall, the increase in EO accumulation under mild and severe water deficit conditions may be due to the decrease in the leaf area of herbs leading to an increase in the density of the glandular trichome per unit of leaf tissue and the production of EO as an osmotic regulator compound [56]. De Abreu and Mazzafera [57] reported that an EO productivity increase under drought stress could be attributed to a low allocation of carbon due to a trade-off between plant defense processes and growth. Rahimzadeh and Pirzad [58] suggested that water deficit can stimulate enzymatic activity for EO biosynthesis. Moreover, the increment of EO contents due to the AMF application may be related to an increased nutrient, especially P, availability in plants by expanding mycorrhiza hyphae [14]. Therefore, improved P uptake of AMF can be postulated as a primary mechanism for the enhanced EO contents in the studied species [48]. The precursors of terpenes (sesquiterpenes and monoterpenes) comprise high-energy phosphate bonds, which require critical cofactors such as NADPH, ATP, and acetyl-CoA for their synthesis. The production of EOs as terpenoids is highly dependent on inorganic phosphorous content; hence, P is an essential limiting factor in the biosynthesis of EOs. P nutrition plays a crucial role in the development and division of the EO channels, secretory ducts, and glandular trichomes. This more significant number of oil glands may be regulated by increasing the level of hormonal profile in plant/AMFs symbiosis, including cytokinins, gibberellins, and auxins [56]. These findings were in agreement with the results of Rydlová et al. [59], who also described an increment of the nutrient concentration and EO content of *Anethum graveolens* from AMF inoculation. In the current study, AMF also promoted the absorption of phosphorous and plant water, which enhanced EO content relative to non-AMF-inoculated samples. The beneficial effect of AMF inoculation on EO accumulation depends on the fungal and host plant species [60]. The effectiveness of AMF in increasing the production of EOs during soil water storage reduction has been illustrated in several aromatic species such as *Pelargonium graveolens* [20], *Foeniculum vulgare* [25,48], *Lavandula officinalis*, *Rosmarinus officinalis*, and *Thymus vulgaris* [61].

Based on the classification of detected terpenes, oxygenated monoterpenes were a major part of EO compositions in *S. abrotanoides* and *S. yangii*. These findings confirm those reported by Ghaffari et al. [5], where the oxygenated monoterpenes were the main constituents of the *S. abrotanoides* EO In the AMF colonized plants these compounds, represented by1,8-cineol, camphor, and borneol, were also elevated disregarding the stress treatment. By contrast, the main sesquiterpenes, including E-β-caryophyllene and α-humulene, remarkably decreased. Prasad et al. [62] and Hazzoumi et al. [21] also suggested that the inoculation of AMF significantly stimulated the content of monoterpenes in the EOs of the *Pelargonium* species and *Ocimum gratissimum*, respectively. AMF symbiosis can improve the biosynthesis of monoterpenes in medicinal plants by regulating phosphorus uptake and improvement of growth attributes, which was finally reflected in EO compositions. The mevalonate pathway (MVA) is the main route for the synthesis of monoterpenes. AMF inoculation might increase the expression of some genes corresponding with monoterpene biosynthesis via the MVA pathway. The mevalonate pathway is necessary for the earliest plant symbiotic signaling produced by AMF. Based on transcriptome analysis of some key enzymes involved in the MVA and methyl erythritol phosphate (MEP) pathways, AMF colonization can elevate isoprenoids through the induction of the MEP pathway. Hence, AMF inoculation can boost the pool of IPP/DMAPP through the MEP pathway. Eventually, AMF symbiosis may reinforce the carbohydrate content followed by the net photosynthesis rate, which is considered the major precursor in monoterpene biosynthesis [47]. Similar to the results of the current study, it has been illustrated that various AMFs under varying intensities can cause changes in EO synthesis in the same plant. In contrast, the same AMF can induce different changes in EO production in distinct plant species. Moreover, water deficit stress can also affect the EO production in plants through beneficial changes in the outcome of symbiosis among the partners [20]. Tarraf et al. [63] highlighted the role of symbiosis with AMFs in medicinal plants belonging to the Lamiaceae family in improving the chemical profile of EOs. The influence of AMF on various constituents of the studied EOs could be related to the change in amounts of various molecules constitutive of EOs, along with enhancing available divalent metallic cations in herbs [59]. Similarly, the beneficial role of AMF inoculation in improving the qualitative and quantitative aspects of EO constituents exposed to water deficit stress has been reported in medicinal plants such as *Thymus daenensis* [12], *Pelargonium graveolens* [20], *Ocimum gratissimum* [21], *Dracocephalum moldavica* [47], and *Cymbopogon citratus* [64].

In the current study, a strong positive correlation was firmly established among E-β-caryophyllene and α-humulene (r = 0.97 **) (Figure 4). Previous reports have revealed the co-presence of α-humulene and E-β-caryophyllene in *S. abrotanoides* [5] and *Salvia* species [62]. The compound 1,8-cineole was also found to be negatively correlated with α-humulene (−0.70 **) and E-β-caryophyllene (0.64 **). A prior study reported similar relationships between 1,8-cineole, α-humulene, and E-β-caryophyllene in the *Perovskia* species [5]. The negative relationship between volatile monoterpenes and sesquiterpenes (Figure 4) could be interpreted as the competitive relationship between two pathways for the same precursor [5]. Clearly, the root colonization percentage was found to be positively correlated with RWC, leaf phosphorus concentration, carotenoid, and chlorophyll *a*. AMF-inoculated plants may inhibit the expression level of the PIPs (plasma membrane intrinsic proteins) gene, thus facilitating water conservation under water deficit conditions in the host herbs [30]. These findings agree with those of Chun-Yan et al. [44] and He et al. [30] in *Camellia sinensis* and *Poncirus trifoliata*, respectively. The obtained significant positive interrelationships among TFC and TPC (r = 0.72 **) can be described by originating polyphenolic compounds from the same biosynthetic route [36]. Moreover, results indicated the significant positive correlations between POD and APX with TFC and TPC (Figure 4), suggesting that polyphenol compounds may play a crucial role as unique substrates for the enzyme production in the network of the enzymatic antioxidant system [36].

According to the PCA dendrogram, a close relation among AMF inoculated with all fungal species followed by a severe stress condition in *S. abrotanoides* and *S. yangii* in group I revealed the important role of root mycorrhizal colonization and reduced soil moisture in rising polyphenol contents and enzymatic activities for EO biosynthesis [46], along with improving the chemical profile of Eos, such as δ-3-carene, as monoterpene hydrocarbon. The physiological characteristics containing chlorophylls *a* and *b*, carotenoid, bornyl acetate, RWC, leaf phosphorus concentration, and root colonization percentage fell into the same category (group II). These results indicated that mycorrhizal symbiosis could significantly improve the absorption of phosphorus, leaf water status, synthesis of photosynthetic pigments, and AMF colonization in plants irrigated optimally in both plant species (group II). Both species exhibited the highest main sesquiterpenes, including E-β-caryophyllene and α-humulene in non-AMF samples, irrespective of irrigation regimes (group III). Considering EO groups, root colonization remarkably decreased the percentage of sesquiterpenes.

## 5. Conclusions

The current study highlighted that essential oil content, enzymatic antioxidant activities, total phenolics, and flavonoids were elevated significantly with the severity of stress; this increase is more pronounced in mycorrhizal inoculated herbs as compared with their control conditions. In addition, leaf phosphorus concentration, relative water content, chlorophylls *a* and *b,* as well as total carotenoids, were diminished in parallel with reducing soil moisture. AMF inoculation alleviated the deleterious impacts of water stress and improved the synthesis of specific secondary metabolites, as well as quality and quantity of Eos, by increasing the phosphorus uptake, chlorophyll biosynthesis, relative water content, and enzymatic and non-enzymatic antioxidant activities. Moreover, with increasing water deficit intensity, *S. yangii,* as a drought-tolerant species, exhibited a better response to AMF inoculation than the other species. Finally, applying AMF symbiosis could be an effective strategy to mitigate the adverse impact of water deficit stress on *S. abrotanoides* and *S. yangii*.

## Figures and Tables

**Table 1:** The origin and taxonomic identification of studied *Salvia* species.

Herbarium Voucher Specimen No.	Species	Location	Longitude	Latitude	Altitude m a.s.l.	Annual Precipitation (mm)
13,368	*S. yangii*	Khash- Sistan and Baluchestan- Iran	28°22′ E	61°18′ N	1410	141.22
13,363	*S. abrotanoides*	Kalat- Laein- Khorasan Razavi- Iran	37°11′ E	59°25′ N	748.8	76.50

**Table 2:** Effect of plant species, AMF inoculation, and water treatments on all biochemical parameters in *Perovskia* species.

Source of Variation	df	TPC	TFC	APX	POD	Phos	RWC	Chl *a*	Chl *b*	Car	EO
Irrigation regime	2	28585.88 **	58.44 **	740680.43 **	4776.20 **	0.04 **	1808.63 **	284.31 **	8.84 **	3.44 **	6.14 **
AMF	3	42646.31 **	65.38 **	319437.26 **	4789.57 **	0.17 **	2074.50 **	254.38 **	14.50 **	1.31 **	6.88 **
Irrigation regime × AMF	6	3111.68 **	0.63 **	41997.87 **	303.01 **	0.01 **	117.45 **	3.73 **	0.44 **	0.05 **	0.28 **
Plant species	1	36802.74 **	26.43 **	137172.74 **	1584.84 **	0.08 **	175.71 **	178.10 **	25.13 **	0.41 **	1.21 **
Irrigation regime × Plant species	2	9015.89 **	7.75 **	25602.60 **	0.64 ^ns^	0.01 **	30.33 **	2.64 **	0.21 **	0.03 **	0.10 **
AMF × Plant species	3	11632.79 **	10.88 **	1682.23 **	93.15 **	0.01 **	14.01 **	10.89 **	0.48 **	0.09 **	0.25 **
Irrigation regime × AMF × Plant species	6	3497.07 **	0.91 **	18158.74 **	62.64 **	0.01 **	2.58 **	4.12 **	0.17 **	0.01 **	0.06 **
Error	48	1.74	0.07	1.18	6.35	0.01	0.46	0.08	0.01	0.01	0.01

^ns^ and **: non-significant and significant at 1% probability levels, respectively; df: degree of freedom.

**Table 3:** The effects of AMF inoculation on all biochemical parameters under different irrigation regimes; TAE: tannic acid; QUE: quercetin; Data are expressed as means ± SD; Different letters indicate significant differences in the irrigation regime × AMF × plant species interaction at *p* ≤ 0.05.

Plant Species	AMF	Irrigation Regime	TPC	TFC	APX	POD	Phos	RWC	Chl *a*	Chl *b*	Car
			(mg TAE g^−1^ DW)	(mg QUE g^−1^ DW)	(μmol min^−1^ mg^−1^ protein)		(mg g^−1^)	(%)	(mg g^−1^ FW)		
*S. abrotanoides*	Non-AMF	WW	94.65 ± 3.62 ^i^	3.35 ± 0.52 ^g^	181.79 ± 7.22 ^j^	8.44 ± 0.91 ^h^	0.37 ± 0.04 ^e^	64.02 ± 1.92 ^c^	10.45 ± 1.17 ^fg^	3.86 ± 0.72 ^d^	1.31 ± 0.14 ^b^
		MWD	111.82 ± 6.11 ^h^	7.71 ± 0.81 ^e^	359.16 ± 10.04 ^g^	14.63 ± 1.28 ^g^	0.35 ± 0.05 ^ef^	53.06 ± 1.05 ^de^	5.57 ± 0.55 ^hi^	3.15 ± 0.39 ^e^	0.83 ± 0.18 ^df^
		SWD	138.25 ± 5.73 ^g^	8.43 ± 0.66 ^d^	486.22 ± 7.51 ^f^	21.41 ± 0.89 ^e^	0.34 ± 0.09 ^ef^	34.90 ± 1.66 ^f^	4.50 ± 0.64 ^i^	2.78 ± 0.11 ^f^	0.76 ± 0.09 ^f^
	*Funneliformis mosseae*	WW	172.17 ± 8.08 ^bc^	9.58 ± 0.75 ^c^	233.26 ± 8.85 ^i^	25.55 ± 1.72 ^d^	0.58 ± 0.11 ^a^	79.24 ± 2.17 ^a^	20.48 ± 1.82 ^a^	6.05 ± 0.96 ^a^	1.98 ± 0.27 ^a^
		MWD	178.90 ± 6.43 ^b^	11.54 ± 0.18 ^b^	775.15 ± 9.27 ^b^	51.39 ± 1.26 ^b^	0.52 ± 0.03 ^b^	75.12 ± 1.33 ^ab^	18.56 ± 1.12 ^b^	5.88 ± 0.93 ^ab^	1.46 ± 0.43 ^b^
		SWD	201.95 ± 7.21 ^a^	12.59 ± 0.95 ^a^	862.67 ± 10.66 ^a^	72.38 ± 1.80 ^a^	0.49 ± 0.10 ^bc^	67.42 ± 2.04 ^bc^	13.34 ± 0.86 ^e^	5.35 ± 0.47 ^b^	1.03 ± 0.07 ^c^
	*Rhizophagus intraradices*	WW	150.47 ± 9.10 ^e^	6.36 ± 0.27 ^f^	381.27 ± 5.13 ^g^	21.35 ± 0.97 ^e^	0.50 ± 0.05 ^b^	77.19 ± 1.70 ^a^	15.48 ± 0.98 ^cd^	4.98 ± 0.39 ^c^	1.68 ± 0.18 ^ab^
		MWD	163.78 ± 5.57 ^de^	9.37 ± 0.81 ^b^	525.82 ± 8.82 ^e^	38.47 ± 1.34 ^c^	0.43 ± 0.03 ^cd^	69.51 ± 1.69 ^b^	9.46 ± 0.33 ^g^	3.67 ± 0.58 ^de^	1.01 ± 0.08 ^c^
		SWD	166.93 ± 6.75 ^cd^	11.20 ± 0.53 ^b^	738.68 ± 11.92 ^bc^	49.39 ± 1.66 ^b^	0.41 ± 0.04 ^d^	63.89 ± 2.28 ^c^	6.69 ± 1.14 ^h^	3.40 ± 0.19 ^de^	0.88 ± 0.08 ^d^
	Mixed AMF	WW	144.79 ± 8.32 ^f^	8.70 ± 0.26 ^d^	325.26 ± 5.07 ^gh^	14.60 ± 0.32 ^g^	0.48 ± 0.04 ^bc^	79.61 ± 1.91 ^a^	16.63 ± 1.76 ^c^	5.40 ± 0.55 ^b^	1.79 ± 0.41 ^a^
		MWD	165.90 ± 9.16 ^cd^	11.27 ± 0.74 ^b^	374.31 ± 8.29 ^g^	19.39 ± 0.28 ^ef^	0.44 ± 0.03 ^c^	71.79 ± 1.88 ^b^	11.75 ± 0.89 ^f^	4.44 ± 0.60 ^c^	1.07 ± 0.07 ^c^
		SWD	180.15 ± 7.37 ^b^	11.80 ± 0.19 ^b^	608.28 ± 7.46 ^d^	40.54 ± 1.58 ^c^	0.37 ± 0.08 ^e^	57.11 ± 0.97 ^d^	9.13 ± 0.27 ^g^	4.25 ± 0.37 ^cd^	0.87 ± 0.12 ^d^
*S. yangii*	Non-AMF	WW	111.22 ± 6.28 ^hi^	7.45 ± 0.27 ^g^	279.28 ± 7.30 ^f^	10.42 ± 0.35 ^g^	0.38 ± 0.08 ^f^	66.60 ± 1.22 ^c^	7.69 ± 0.32 ^de^	2.91 ± 0.18 ^c^	1.01 ± 0.08 ^bc^
		MWD	124.23 ± 4.91 ^h^	8.07 ± 0.96 ^f^	401.01 ± 9.92 ^e^	17.29 ± 0.92 ^f^	0.36 ± 0.05 ^f^	51.62 ± 1.68 ^d^	3.49 ± 0.65 ^g^	2.61 ± 0.29 ^cd^	0.65 ± 0.08 ^e^
		SWD	187.13 ± 8.47 ^d^	9.35 ± 0.25 ^e^	529.80 ± 5.69 ^d^	25.52 ± 1.08 ^e^	0.35 ± 0.05 ^fg^	40.07 ± 1.91 ^e^	2.85 ± 0.14 ^gh^	1.86 ± 0.44 ^f^	0.38 ± 0.04 ^f^
	*Funneliformis mosseae*	WW	171.60 ± 8.12 ^df^	11.77 ± 0.61 ^c^	505.61 ± 7.17 ^d^	38.30 ± 1.01 ^d^	0.72 ± 0.09 ^a^	79.43 ± 1.96 ^ab^	17.01 ± 0.45 ^a^	4.85 ± 0.42 ^a^	1.78 ± 0.39 ^a^
		MWD	351.03 ± 10.64 ^b^	12.30 ± 0.11 ^b^	638.93 ± 8.85 ^c^	55.38 ± 0.79 ^b^	0.68 ± 0.04 ^a^	76.77 ± 2.08 ^b^	10.52 ± 1.16 ^c^	4.52 ± 0.85 ^a^	1.45 ± 0.11 ^ab^
		SWD	393.97 ± 9.73 ^a^	14.32 ± 0.58 ^a^	1044.66 ± 11.39 ^a^	86.38 ± 1.86 ^a^	0.59 ± 0.11 ^b^	72.77 ± 1.77 ^bc^	8.47 ± 0.70 ^d^	3.11 ± 0.17 ^bc^	1.04 ± 0.08 ^bc^
	*Rhizophagus intraradices*	WW	126.09 ± 6.68 ^h^	10.36 ± 0.82 ^d^	536.58 ± 4.22 ^d^	37.73 ± 1.55 ^d^	0.61 ± 0.08 ^b^	77.90 ± 2.14 ^b^	11.83 ± 0.85 ^bc^	3.95 ± 0.63 ^b^	1.29 ± 0.09 ^b^
		MWD	198.69 ± 9.45 ^cd^	11.66 ± 0.37 ^c^	610.64 ± 8.39 ^c^	47.40 ± 1.87 ^c^	0.55 ± 0.09 ^c^	71.07 ± 1.55 ^bc^	6.48 ± 0.19 ^e^	2.62 ± 0.10 ^cd^	0.81 ± 0.12 ^d^
		SWD	211.24 ± 5.07 ^c^	12.51 ± 0.44 ^b^	786.60 ± 7.09 ^b^	56.44 ± 1.45 ^b^	0.48 ± 0.05 ^d^	68.31 ± 1.69 ^c^	5.50 ± 0.62 ^f^	2.14 ± 0.08 ^e^	0.70 ± 0.08 ^de^
	Mixed AMF	WW	158.99 ± 7.82 ^g^	8.47 ± 0.18 ^f^	433.80 ± 7.61 ^e^	21.45 ± 0.79 ^e^	0.51 ± 0.06 ^c^	84.17 ± 1.31 ^a^	12.46 ± 0.48 ^b^	3.81 ± 0.97 ^b^	1.71 ± 0.24 ^a^
		MWD	182.24 ± 5.33 ^d^	9.64 ± 0.71 ^e^	503.81 ± 8.55 ^d^	42.37 ± 1.33 ^c^	0.47 ± 0.08 ^d^	76.62 ± 1.08 ^b^	10.68 ± 0.75 ^c^	3.45 ± 0.46 ^b^	1.05 ± 0.09 ^bc^
		SWD	195.93 ± 8.49 ^cd^	10.54 ± 0.59 ^d^	628.72 ± 9.12 ^c^	51.44 ± 1.68 ^b^	0.42 ± 0.04 ^de^	65.01 ± 1.28 ^c^	7.32 ± 0.66 ^de^	3.21 ± 0.15 ^bc^	0.99 ± 0.05 ^bc^

**Table 4:** The effects of AMF inoculation on essential oil chemical compositions and relative content (%) of *S. abrotanoides* under different irrigation regimes.

Compounds	RI ^a^	Non-AMF			*Funneliformis mosseae*			*Rhizophagus intraradices*			Mixed AMF		
		WW	MWD	SWD	WW	MWD	SWD	WW	MWD	SWD	WW	MWD	SWD
α-Pinene	937	nd ^b^	2.90	3.85	1.09	4.89	3.99	1.12	5.88	5.51	2.18	2.60	5.67
Camphene	951	nd	1.11	1.40	1.05	3.99	3.28	1.06	4.66	4.66	2.03	2.37	4.55
β-Pinene	973	nd	0.18	0.22	nd	0.49	0.61	nd	0.47	0.48	nd	nd	0.59
Myrcene	991	0.79	1.15	1.11	1.01	1.52	0.94	1.12	2.00	2.44	1.27	1.35	2.40
**δ-3-Carene**	**1011**	**1.91**	**0.15**	**0.70**	**4.27**	**7.40**	**6.90**	**3.60**	**7.93**	**8.16**	**5.19**	**6.95**	**7.34**
*p*-Cymene	1025	nd	0.38	0.95	nd	0.85	0.76	nd	0.95	1.07	0.61	0.77	1.02
Limonene	**1030**	0.75	1.05	1.85	0.91	2.01	1.24	1.01	1.43	1.60	1.15	1.20	1.58
**1,8-Cineole**	**1032**	**5.20**	**9.41**	**12.77**	**11.15**	**17.34**	**19.33**	**11.50**	**19.11**	**20.09**	**12.63**	**17.13**	**20.34**
Linalool	1099	nd	0.82	0.40	nd	0.57	nd	nd	nd	nd	nd	nd	nd
**Camphor**	**1142**	**10.13**	**9.90**	**15.88**	**18.82**	**23.16**	**19.34**	**17.62**	**15.48**	**17.66**	**21.66**	**16.69**	**23.53**
*cis*-Chrysanthemol	1160	nd	1.45	0.65	nd	0.51	nd	nd	nd	0.52	nd	nd	0.50
**Borneol**	**1167**	**5.34**	**4.77**	**3.20**	**5.46**	**4.88**	**11.60**	**11.92**	**11.03**	**8.78**	**7.55**	**6.71**	**5.05**
Terpinen-4-ol	1177	nd	nd	1.69	nd	0.49	nd	nd	nd	nd	nd	nd	nd
Myrtenol	1195	nd	0.71	0.13	0.78	0.81	1.09	0.78	0.98	1.04	0.74	1.11	0.79
**Bornyl acetate**	**1285**	**7.65**	**4.18**	**2.10**	**5.05**	**3.08**	**8.43**	**10.23**	**5.93**	**4.25**	**4.82**	**7.17**	**3.89**
α-Terpinyl acetate	1350	3.66	1.60	1.10	2.67	1.73	2.04	2.87	1.47	1.43	2.25	1.99	1.67
Geranyl acetate	1382	nd	1.65	2.12	nd	nd	nd	0.55	nd	nd	nd	nd	nd
α-Gurjunene	1409	0.82	2.92	1.10	0.70	nd	nd	0.72	nd	0.62	nd	nd	0.45
**E-β-Caryophyllene**	**1421**	**8.97**	**7.19**	**6.50**	**7.98**	**6.41**	**3.93**	**5.71**	**4.69**	**4.52**	**6.51**	**5.79**	**4.64**
**α-Humulene**	**1454**	**8.02**	**7.44**	**6.91**	**6.80**	**5.19**	**3.26**	**4.83**	**4.10**	**3.65**	**5.84**	**4.88**	**3.82**
*allo*-aromadendrene	1461	0.60	2.31	1.44	1.86	nd	nd	0.77	nd	nd	2.41	nd	nd
γ-Cadinene	1513	2.24	2.18	1.55	1.74	0.68	0.91	1.27	0.73	1.27	1.47	1.31	0.96
δ-Cadinene	1524	1.81	1.24	0.90	1.18	0.47	0.54	0.93	0.68	0.55	0.76	0.55	0.62
Spathulenol	1576	nd	1.46	2.31	nd	nd	0.49	0.58	0.46	0.50	nd	nd	0.38
Caryophyllene oxide	1581	1.58	0.77	0.34	1.15	0.61	0.49	1.01	2.83	nd	1.14	0.96	0.56
Viridiflorol	1591	0.89	2.35	2.33	0.62	nd	nd	nd	nd	nd	nd	0.53	nd
β-Oplopenone	1606	1.61	2.11	1.65	0.87	nd	0.50	0.68	nd	0.65	0.90	0.81	nd
Isomyristicin	1615	0.91	3.29	3.02	0.69	nd	nd	nd	nd	nd	1.05	nd	nd
Calarene	1620	1.98	3.25	2.30	1.48	0.84	0.71	1.30	0.67	0.61	1.48	1.28	0.72
*tau*-Cadinol	1640	8.89	6.11	5.20	4.54	1.75	2.30	3.45	1.73	3.22	4.68	4.34	2.29
t-Muurolol	1642	6.11	5.29	4.12	4.49	2.79	2.41	4.11	2.41	2.39	3.47	3.89	2.34
Fonenol	1648	1.30	0.37	0.91	1.08	2.08	nd	0.78	nd	1.32	0.88	0.81	1.62
α-Eudesmol	1653	2.29	0.95	1.81	1.07	nd	0.49	0.82	0.72	nd	0.78	0.52	nd
α-Cadinol	1654	5.80	4.22	2.88	3.92	nd	1.52	3.38	1.45	nd	2.73	3.11	nd
β-Bisabolole	1673	4.16	3.74	2.50	2.95	1.79	1.22	2.34	1.42	1.26	2.32	2.33	1.38
α-Bisabolol	1684	4.06	2.98	3.21	4.04	3.09	1.70	3.96	0.78	1.75	1.49	2.86	1.29
Monoterpene hydrocarbons		3.45	6.92	10.08	8.33	21.15	17.72	7.91	23.32	23.92	12.43	15.24	23.15
Oxygenated monoterpenes		20.67	27.06	34.72	36.21	47.76	51.36	41.82	46.60	48.09	42.58	41.64	50.21
Sesquiterpene hydrocarbons		22.46	23.28	18.40	20.26	12.75	8.64	14.23	10.20	10.61	16.99	12.53	10.49
Oxygenated sesquiterpenes		38.67	33.60	29.56	26.21	12.95	11.83	22.41	12.47	11.70	19.87	21.44	10.58
Others		12.22	9.14	7.24	99.42	4.81	10.45	13.63	7.40	5.68	8.12	9.15	5.56
Total identified		97.47	100	100	99.43	99.42	100	100	99.99	100	99.99	100	99.99
Essential oil content (%)		0.54	0.82	1.70	1.87	2.56	3.78	1.54	1.88	2.20	1.76	2.07	2.38

Bold values show the main components. ^a^ RI: retention indices (RIs) as determined by a DB-5 MS column using a homologous series of *n*-alkanes (C_8_–C_24_). ^b^ nd: not detected.

**Table 5:** The effects of AMF inoculation on essential oil chemical compositions and relative content (%) in *S. yangii* under different irrigation regimes.

Compounds	RI ^a^	Non-AMF			*Funneliformis mosseae*			*Rhizophagus intraradices*			Mixed AMF		
		WW	MWD	SWD	WW	MWD	SWD	WW	MWD	SWD	WW	MWD	SWD
α-Pinene	937	1.79	2.08	3.45	1.91	3.40	7.38	1.95	3.53	4.85	4.86	5.67	5.82
Camphene	951	1.47	1.30	2.15	1.49	2.78	4.72	1.63	3.05	3.70	3.68	4.36	4.22
β-Pinene	973	nd ^b^	1.89	1.93	0.60	0.90	1.04	nd	0.57	0.56	1.08	1.01	1.27
Myrcene	991	0.76	2.95	3.26	0.83	0.79	0.98	1.03	0.97	1.69	0.71	0.70	1.18
**δ-3-Carene**	**1011**	**2.57**	**4.89**	**4.40**	**2.88**	**5.29**	**10.21**	**2.82**	**5.90**	**6.21**	**6.51**	**6.27**	**6.43**
*p*-Cymene	1025	nd	0.88	0.37	0.70	0.70	1.30	nd	0.51	0.93	0.98	1.16	0.98
Limonene	**1030**	3.22	2.19	3.24	2.47	2.62	6.00	0.99	1.52	3.97	5.59	5.32	7.07
**1,8-Cineole**	**1032**	**14.53**	**14.33**	**17.67**	**15.81**	**24.91**	**26.82**	**11.54**	**21.52**	**23.91**	**24.24**	**25.36**	**30.20**
Linalool	1099	nd	1.52	2.39	nd	nd	nd	nd	1.27	nd	nd	nd	0.47
**Camphor**	**1142**	**4.44**	**8.25**	**2.95**	**6.49**	**14.64**	**2.68**	**15.10**	**13.37**	**13.88**	**11.95**	**15.21**	**3.52**
*cis*-Chrysanthemol	1160	nd	2.34	4.21	nd	0.65	nd	nd	0.78	0.53	0.63	0.74	0.76
**Borneol**	**1167**	**4.64**	**2.87**	**3.63**	**6.61**	**8.35**	**8.48**	**6.55**	**5.46**	**6.45**	**5.19**	**4.58**	**6.76**
Terpinen-4-ol	1177	nd	0.29	0.24	nd	0.57	nd	nd	nd	0.50	0.53	0.66	0.52
Myrtenol	1195	1.02	1.71	1.58	0.75	0.81	0.75	0.55	1.34	0.72	0.88	0.91	0.96
**Bornyl acetate**	**1285**	**9.92**	**4.52**	**3.66**	**12.93**	**8.50**	**10.52**	**12.02**	**5.84**	**7.13**	**7.71**	**6.96**	**10.96**
α-Terpinyl acetate	1350	3.36	2.01	1.83	3.90	3.33	2.46	4.21	2.54	2.79	3.03	2.71	2.94
α-Gurjunene	1409	0.70	0.82	0.45	0.73	nd	nd	0.70	nd	0.57	nd	nd	0.29
**E-β-Caryophyllene**	**1421**	**11.42**	**7.07**	**6.14**	**8.59**	**5.87**	**4.49**	**8.70**	**6.41**	**4.62**	**4.66**	**5.37**	**5.15**
endo Bornyl acetate	1448	nd	0.21	0.15	1.06	nd	nd	nd	nd	nd	nd	nd	nd
**α-Humulene**	**1454**	**10.39**	**6.91**	**5.45**	**7.24**	**4.94**	**3.66**	**7.25**	**5.59**	**3.81**	**4.09**	**4.45**	**3.82**
allo-aromadendrene	1461	4.15	1.18	1.44	10.07	2.05	2.15	2.53	3.43	3.05	1.57	2.81	3.46
Calamenene	1497	0.65	2.37	2.45	0.80	nd	nd	nd	nd	nd	nd	nd	nd
γ-Cadinene	1513	1.23	1.19	1.78	0.70	0.82	0.66	1.57	0.60	0.99	1.07	0.53	0.31
δ-Cadinene	1524	2.08	1.87	1.12	nd	0.56	0.34	1.11	nd	0.63	0.41	0.41	nd
Caryophyllene oxide	1581	1.42	0.75	0.32	1.23	0.83	0.42	0.75	1.05	0.51	0.91	0.50	0.37
Viridiflorol	1591	0.73	1.63	1.25	nd	nd	nd	0.91	0.57	nd	nd	nd	nd
β-Oplopenone	1606	nd	1.82	1.25	nd	nd	0.32	0.97	nd	nd	0.62	nd	nd
Isomyristicin	1615	1.00	0.28	0.19	2.17	0.48	0.42	1.20	0.84	0.74	0.49	0.45	0.49
Calarene	1620	1.70	0.40	0.18	1.55	0.95	0.54	1.15	1.36	0.66	1.08	0.64	0.31
*tau*-Cadinol	1640	1.94	1.91	0.18	2.04	2.18	1.61	5.64	1.72	2.34	3.40	0.99	0.71
t-Muurolol	1642	3.52	4.48	5.63	1.91	1.02	0.83	3.14	2.75	1.76	1.55	0.74	0.56
Fonenol	1648	1.49	3.14	2.38	1.85	nd	0.68	nd	2.23	1.38	1.35	nd	nd
α-Eudesmol	1653	2.93	3.27	3.01	nd	0.51	nd	1.07	nd	nd	nd	0.44	nd
α-Cadinol	1654	3.62	1.89	2.28	nd	0.91	nd	2.29	nd	nd	nd	0.62	nd
β-Bisabolole	1673	3.29	1.27	2.07	2.02	0.66	0.54	2.11	1.63	1.13	1.22	0.43	nd
α-Bisabolol	1684	nd	1.62	0.18	nd	nd	nd	nd	3.03	nd	nd	nd	nd
Monoterpene hydrocarbons	-	9.81	24.18	26.80	10.88	16.48	30.33	8.42	16.05	21.91	23.41	24.49	26.97
Oxygenated monoterpenes	-	24.63	31.31	32.67	29.66	49.93	38.73	33.74	43.74	45.99	43.42	47.46	43.19
Sesquiterpene hydrocarbons	-	28.54	11.54	9.71	28.13	13.68	10.96	20.75	16.03	13.04	11.39	13.16	13.03
Oxygenated sesquiterpenes	-	22.72	24.05	19.85	10.60	7.62	5.28	19.14	14.34	8.41	10.54	4.77	1.95
Others	-	14.28	7.02	5.83	20.06	12.29	13.40	17.43	9.22	10.65	11.23	10.12	14.39
Total identified	-	99.98	98.10	94.86	99.30	100	98.70	99.48	99.38	100	99.99	100	99.53
Essential oil content (%)		0.61	0.94	1.39	1.58	2.36	2.90	1.37	1.86	2.17	1.16	1.63	2.01

Bold values show the main components. ^a^ RI: retention indices (RIs) as determined by a DB-5 MS column using a homologous series of *n*-alkanes (C_8_–C_24_). ^b^ nd: not detected.

## Data Availability

Data contained within the article are available upon reasonable request from the corresponding author (M.R.).

## Abbreviations

AMF	arbuscular mycorrhizal fungi
TPC	total phenolic content
TFC	total flavonoid content
APX	ascorbate peroxidase
POD	guaiacol peroxidase
Phos	phosphorus concentration
RWC	relative water content
Chl *a*	chlorophyll *a*
Chl *b*	chlorophyll *b*
Car	carotenoid
EO	essential oil content
WW	well-watered
MWD	moderate drought stress
SWD	severe drought stress
